# Cloning, expression and antioxidant activity of a thioredoxin peroxidase from *Branchiostoma belcheri tsingtaunese*

**DOI:** 10.1371/journal.pone.0175162

**Published:** 2017-04-06

**Authors:** Jian Liao, Kaiyu Wang, Weirong Yao, Xunfei Yi, Huihui Yan, Min Chen, Xiaopeng Lan

**Affiliations:** 1 Institute for Laboratory Medicine, Fuzhou General Hospital of Nanjing Command, Fuzhou, Fujian Province, China; 2 Clinical Laboratory, The First Hospital of Longhai, Zhangzhou, Fujian Province, China; Laboratoire Arago, FRANCE

## Abstract

Peroxiredoxins (Prxs) are ubiquitous antioxidant enzymes that catalyze the thioredoxin- dependent reduction of hydroperoxides. In this study, a novel thioredoxin peroxidase (Bbt-TPx1), a member of the peroxiredoxin superfamily, was found by EST sequence analysis of a cDNA library of *Branchiostoma belcheri tsingtaunese* ovary. The sequence of a full-length cDNA clone contained an open reading frame encoding a polypeptide of 198 amino acid residues, with a calculated molecular weight of 22,150 Da. The expression patterns of the protein at different developmental stages and adult amphioxus tissues indicate that this enzyme may play important roles in anti-oxidation and innate immunity. The recombinant Bbt-TPx1 protein was expressed with a polyhistidine-tag in *Escherichia coli* and purified using Ni chromatography followed by SP cation exchange chromatography. The rBbt-TPx1 protein existed as a dimer under non-reducing conditions, and was dissociated into monomers by dithiothreitol (DTT); it might predominantly exist in oligomeric form. The rBbt-TPx1 protein showed a significant thiol-dependent peroxidase activity, removing hydrogen peroxide in the presence of dithiothreitol (DTT), but not glutathione (GSH). Protection of plasmid DNA and the thiol-protein from damage by metal-catalyzed oxidation (MCO) *in vitro* was also revealed.

## Introduction

Reactive oxygen species (ROS) are constantly generated under normal conditions as a consequence of aerobic metabolism. ROS include free radicals such as superoxide anions (O_2_·–), hydroxyl radicals (HO·), and non-radical hydrogen peroxide (H_2_O_2_). They are transient species due to their chemical reactivity, and react with DNA, proteins and lipids in a destructive manner [1]. To protect themselves from the deleterious effects of ROS, cells have developed a wide range of antioxidant systems. However, oxidative stress may occur when the balance between ROS production and antioxidant defense is disrupted. To survive, aerobic organisms are equipped with several antioxidant enzymes, which can remove the harmful ROS [2].

Thioredoxin peroxidase (TPx), a member of the peroxiredoxin (Prx) gene superfamily, was first reported in *Saccharomyces cerevisiae* [3]. TPx functions as an antioxidant to remove O_2_·– and H_2_O_2_ derived from normal cellular metabolism, using thioredoxin as the electron donor [4].

Instead of using metals or other redox cofactors, TPx has a distinctive Cys residue at the peroxide reduction site, a feature that distinguishes this enzyme from other conventional peroxidases [3]. A group of peroxidases containing the distinctive Cys residue have been grouped in the peroxiredoxin (Prx) family instead of the TPx family, because not all members of this family use thioredoxin as immediate electron donor [5]. The members of this family have been identified with multiple isotypes from all kingdoms, from Archaebacteria and humans. TPx, in presence of the Trx system, NADPH, thioredoxin reductase (TR) and Trx, constitutes one of the major pathways that remove toxic peroxides from cells. Hydrogen peroxide, a toxic byproduct of cellular respiration, is also believed to be a second messenger of cellular signaling cascade [6]. Provided hydrogen peroxide (second messenger) and Trx (a well-known growth mediator), TPx, characterized by very high affinity to peroxides, is widely considered a signal modulator as well as an antioxidant [7]. Either functioning as signal modulator or antioxidant, regulation of its activity is necessary for accurate and rapid signal transmission, and to maintain the redox balance of the cell. In addition, Prx has been implicated in oxidative signalling mechanisms regulating cell apoptosis, differentiation, and proliferation [8].

Amphioxus is considered to be a transitional species between the invertebrates and vertebrates, of which the genome may be at the initial point of early vertebrates' massive gene duplication [9]. Previous genetic studies have proved that amphioxus owns typical invertebrate genome feature [10]. Therefore, amphioxus could be an ideal model for evolutionary studies since it possesses genes that are directly orthologous to invertebrates. Besides, its genes may also be comparable with those of vertebrates, making it possible to deduce specific gene history (i.e. duplication timing and functional projection) [11,12].

In this study, we described cDNA cloning and identification of a novel thioredoxin peroxidase (Bbt-TPx1) from the Chinese amphioxus, *Branchiostoma belcheri tsingtaunese*, in which there are at least three Prx genes. In addition, amphioxus TPx1 transcription patterns were assessed at different developmental stages and adult tissues. Finally, structure and enzymological properties of the recombinant Bbt-TPx1 protein produced in *Escherichia coli* were evaluated.

## Material and methods

### Ethics statement

All animal experiments were conducted in accordance with the guidelines and approval of the Animal Research and Ethics Committees of Fuzhou General Hospital of Nanjing Command. All efforts were made to minimize suffering.

### Animal fishing

The Chinese amphioxus, *Branchiostoma belcheri tsingtaunese* (genus Branchiostoma, family Branchiostomidae) individuals were obtained from Kioachow Bay near Qingdao, China. *Branchiostoma belcheri tsingtaunese* were separated from sea mud with a specific kind of screen mesh. Before sample collection, the *Branchiostoma belcheri tsingtaunese* were anaesthetized with MS-222 in dechlorinated water for 2 min followed by sacrifice by immersion in liquid nitrogen.

### cDNA library generation

The *Branchiostoma belcheri tsingtaunese* ovaries were frozen with liquid nitrogen quickly and triturated into powder. Then total RNA was extracted from ovaries by the TRIzol^®^ LS Reagent (Gibco BRL), and cDNA library constructed using the SMART^™^ cDNA Construction Kit (Clontech).

### Identification of TPx1 cDNA

EST sequences obtained from *Branchiostoma belcheri tsingtaunese* ovary cDNA library were analyzed for possible encoded functional proteins. Sequence homology comparisons of each EST were performed against the GenBank database using BLASTX algorithms, followed by BLASTP searches for open reading frames. ESTs with homology to previously identified sequences were given tentative names based on homologous sequence names. Using this method, a TPx1 EST was identified, and the TPx1 cDNA was found to be full-length.

### Amino acid sequence alignment and phylogenetic tree construction

To study the evolutionary status of Bbt-Tpx1, protein sequences of Prx/Tpx genes with two typical cys residues from various vertebrate species were collected from NCBI GenBank and Ensembl. Exceptionally, hypothetical Prx amino acids of *Branchiostoma floridae* were also found in NCBI GenBank (XP_002586269.1 and XP_002605995.1), while amino acid sequences of Prx genes of *Branchiostoma belcheri* were collected from http://genome.bucm.edu.cn/lancelet/index.php. The predicted protein sequences of Bbt-TPx1 and Prx1/Prx2/TPx from other free-living organisms were aligned using CLUSTAL Omega (http://www.ebi.ac.uk/Tools/msa/clustalo/), with Prx motifs highlighted in grey and cys residues highlighted in pink. All Prxs with two typical cys residues (Prx1-4) and Tpxs were aligned and constructed into a Maximum Likelihood phylogenetic tree using MEGA6, with a bootstrap of 100 times to verify its credibility.

### Real-Time Polymerase Chain Reaction (RT-PCR)

Total RNA samples from *Branchiostoma belcheri tsingtaunese* tissues at different developmental stages and adulthood were obtained with TRIzol Reagent (Gibco BRL), according to the manufacturer’s instructions. Then, cDNA was synthesized using SMART^™^ cDNA library construction kit (CLONTECH), following the protocol for first-strand cDNA synthesis provided by the manufacturer. Parallel reactions were performed without reverse transcriptase to monitor possible genomic DNA contamination. PCR reactions were then performed with the up-stream primer 5’-CGC GGA TCC TCT GCT GGA AAT GCC AAG-3’ and down- stream primer 5’-CCG CTC GAG TCA TTA CTG CTT GGA GAA ATA TTC TTT G-3’ to amplify a 600-nucleotide (nt) fragment containing the Bbt-TPx1 cDNA. A primer pair of *Branchiostoma belcheri tsingtaunese* β-actin (AC-F: 5’-CAG CTA CGT CGG TGA TGA GG-3’ and AC-R: 5’-GCT TCT CCT TGA TGT CAC GG-3’) was used to amplify a control 500 nt fragment in each sample.

### Construction of the Bbt-TPx1 expression vector pET32a (+) M-Bbt-TPx1

The open reading frame of the cDNA sequence encoding the mature TPx1 was amplified by PCR using the upstream primer (with *BamHI* site) 5'-CGC GGA TCC TCT GCT GGA AAT GCC AAG–3' and downstream primer (with *XhoI* site) 5'-CCG CTC GAG TCA TTA CTG CTT GGA GAA ATA TTC TTT G–3'. The PCR product was digested with *BamHI* and *XhoI*, followed by ligation into the pET32a (+) M vector (Novagen), to yield the expression plasmid pET32a (+) M-Bbt-TPx1, which produced the Bbt-TPx1 protein with an N-terminal His-Tag. Positive clones were sequenced with a BigDye^™^ DNA Sequencing Kit (Applied Biosystems) to ensure they contained no mutations.

### Expression and purification of the recombinant Bbt-TPx1

The original pET32a (+) M vector as well as recombinant plasmid (pET32a (+) M—Bbt-TPx1) were transformed into *E*. *coli* strain BL21 (DE3)/pLysS, and propagated in Luria broth (LB) containing 100 μg/ml ampicillin for selection. Recombinant protein expression was induced in exponentially growing bacteria (*A*600 = 0.6–0.8) with 0.1 mM isopropyl- 1-thio–b-D- galactopyranoside for 10 h at 20°C with vigorous agitation. Then, bacteria were harvested by centrifugation, washed with TE buffer (10 mM Tris, 1 mM EDTA, pH 8.0) and resuspended in lysis buffer (50 mM Tris-HCl, pH 8.0) containing 500 mM NaCl. Cells were lysed on ice by mild sonication, and the lysate centrifuged at 5,000 rpm for 30 min. All subsequent purification steps were carried out at 25°C. The cleared lysate (supernatant) was applied onto a Ni^2+^ chelate column (Pharmacia Biotech) equilibrated with lysis buffer. After washing the column with the same buffer, the bound protein was eluted with 50 mM Tris-HCl (pH 8.0) containing 500 mM NaCl and a series of increasing imidazole concentrations. All fractions were submitted to SDS-PAGE, and gels were stained with Coomassie Blue R-250 for analysis. The fusion protein purified with the Ni^2+^ chelate column was applied to a SP-Sepharose column (Pharmacia Biotech) equilibrated with 50 mM PB, pH 6.3. After washing the column with equilibration buffer, the bound protein was eluted with 50 mM PBS (pH 7.0) containing 500 mM NaCl. Purity of Bbt-TPx1 was assessed by SDS-PAGE, and protein concentrations were determined by the Lowry Protein Quantitation Kit (Biocolor Co. Ltd.), using a standard curve generated with BSA.

### Non-reducing SDS-PAGE analysis

Fractionation by SDS-PAGE was performed on a 12% (w/v) polyacrylamide gel as described by Laemmli Protocol. For SDS-PAGE under non-reducing conditions, DTT was omitted from the loading buffer. Proteins bands were stained with Coomassie brilliant blue R-250.

### Analysis by gel filtration chromatography

The rBbt-TPx1 protein was analyzed by gel filtration on a ODS-3 column (DIKMA) (6 mm*250 mm).

### *In vitro* enzyme activity assay

The antioxidant activity of rBbt-TPx1, reflected by H_2_O_2_ removal, was assessed as described by Lim *et al*. [2], with the following modifications. Reaction mixtures (1 ml) containing 50 mM HEPES (pH 7.0) and rBbt-TPx1 (20–100 μg/ml) were pre-incubated with or without 5 mM dithiothreitol (DTT) or 5 mM glutathione (GSH) at room temperature for 10 min. Afterwards, H_2_O_2_ (200 μM) was added for 10 min. The reaction was stopped by addition of 10% (v/v) trichloroacetic acid. H_2_O_2_ remaining in the reaction mixture reacted with 200 μl of 10 mM Fe (NH_4_)_2_(SO_4_)_2_ and 100 μl of 2.5 M KSCN (per 1 ml reaction mixture) to produce the ferrithiocyanate complex, whose red color was measured by colorimetry at 475 nm.

Metal-catalyzed oxidation (MCO) DNA cleavage protection assays were performed essentially as described by Sauri *et al* [13] with a few modifications. Reaction mixtures containing 3 μM FeCl_3_, 10 mM dithriothreitol (DTT) and the rBbt-TPx1 protein (25–500 μg/ml) were incubated in a total volume of 50 μl at 37°C. After 2h, 1000 ng of pGAPZαA supercoiled plasmid DNA was added to each reaction for 2.5h at 37°C. DNA degradation was assessed by electrophoresis on a 0.8% (w/v) agarose gel containing 0.5 μg/ml ethidium bromide.

rBbt-TPx1 antioxidant activity was also assayed by monitoring the ability to inhibit the thiol/Fe^3+^/O_2_-mediated inactivation of GS as described by Kim *et al* [3] with a few modifications. Assays were performed in 50 μl reaction mixtures containing 10 μg/ml of GS, 10 mM DTT, 3 μM FeCl_3_, 50 mM imidazole–HCl, and the rBbt-TPx1 protein (20–100 μg/ml) at pH 6.7. Control reaction mixtures with 1 mM EDTA (positive control) or without the rBbt-TPx1 protein (negative control) were also setup. All mixtures were incubated at 30°C for 25 min. At 0, 2, 8, 15, and 25 min, residual GS activity was measured by addition of 2 ml of γ-glutamyl transferase assay mixture containing 0.4 mM ADP, 150 mM glutamine, 10 mM KAsO_4_, 20 mM NH_2_OH, 0.4 mM MnCl_2_, and 100 mM HEPES (pH 7.4). Reactions were carried out at 30°C for 5 min and terminated by addition of 1 ml of a stop mixture (64 g FeCl_3_, 100 g trichloroacetic acid, and 41 ml of concentrated HCl/liter). Absorbance of the γ- glutamyl transferase–Fe^3+^ complex was measured at 540 nm.

## Results and discussion

### Sequence analysis of Bbt-TPx1

**Fig 1:**
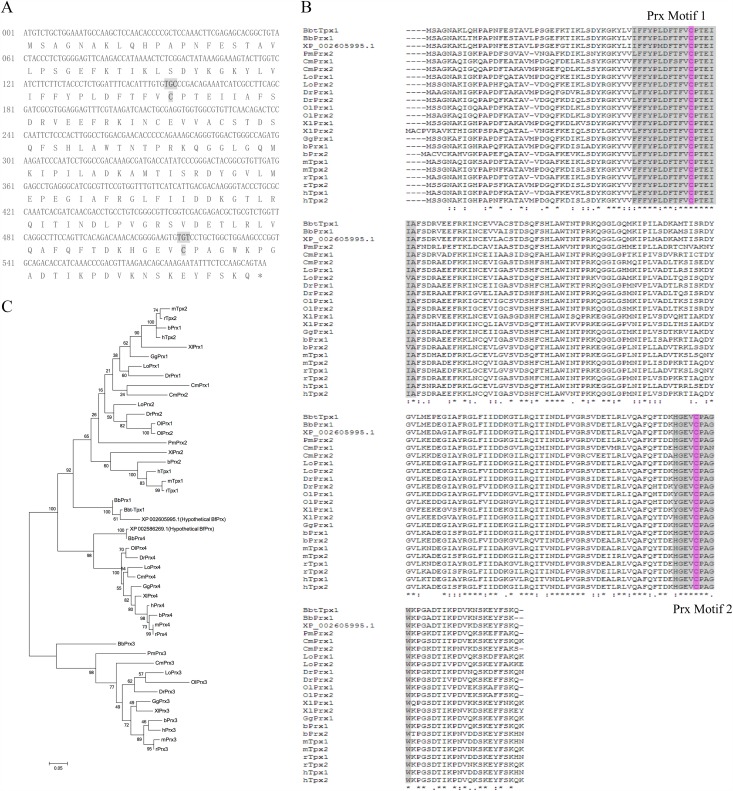
Sequence analysis of Bbt-TPx1. (A) Nucleotide and deduced amino acid sequences of the cDNA encoding Bbt-TPx1. Active sites are shown in bold. (B) Alignment of amino acid sequences of Bbt-TPx1 with other Family 1, 2 Prx members from various species. Prx motifs were highlighted in grey and the typical cys residues were highlighted in pink. (C) Maximum Likelihood (ML) phylogenetic tree relating Bbt-TPx1 with other Prx members with two typical cys residues (Prx1-4) from various species. Amino acids were mainly collected form NCBI with a few exceptions and were listed as follows: Bbt-Tpx1(*Branchiostoma belcheri tsingtaunese* Tpx1, AAU84951.1), hTpx1(*Homo sapiens* Tpx1, NP_005800.3), hTpx2(*Homo sapiens* Tpx2, NP_001189360.1), hPrx3(*Homo sapiens* Prx3, NP_001289201.1), hPrx4(*Homo sapiens* Prx4, NP_006397.1), mTpx1(*Mus musculus* Tpx1, NP_001304314.1), mTpx2(*Mus musculus* Tpx2, NP_035164.1), mPrx3(*Mus musculus* Prx3, NP_031478.1), mPrx4(*Mus musculus* Prx4, NP_001300640.1), rTpx1(*Rattus norvegicus* Tpx1, NP_058865.1), rTpx2(*Rattus norvegicus* Tpx2, NP_476455.1), rPrx3(*Rattus norvegicus* Prx3, EDL94585.1), rPrx4(*Rattus norvegicus* Prx4, NP_445964.1), bPrx1(*Bos taurus* Prx1, NP_776856.1), bPrx2(*Bos taurus* Prx2, NP_777188.1), bPrx3(*Bos taurus* Prx3, NP_776857.1), bPrx4(*Bos taurus* Prx4, NP_776858.1), GgPrx1(*Gallus gallus* Prx1, NP_001258861.1), GgPrx3(*Gallus gallus* Prx3, XP_426543.4), GgPrx4(*Gallus gallus* Prx4, XP_416800.2), XlPrx1(*Xenopus laevis* Prx1, NP_001011135.1), XlPrx2(*Xenopus laevis* Prx2, NP_989001.1), XlPrx3(*Xenopus laevis* Prx3, NP_001025608.1), XlPrx4(*Xenopus laevis* Prx4, NP_001006812.1), DrPrx1(*Danio rerio* Prx1, NP_001013489.2), DrPrx2(*Danio rerio* Prx2, NP_001002468.1), DrPrx3(*Danio rerio* Prx3, NP_001013478.3), DrPrx4(*Danio rerio* Prx4, NP_001082894.1), OlPrx1(*Oryzias latipes* Prx1, AEA51065.1), OlPrx2(*Oryzias latipes* Prx2, XP_011478977.1), OlPrx3(*Oryzias latipes* Prx3, XP_011483095.1), OlPrx4(*Oryzias latipes* Prx4, XP_004076406.1), LoPrx1(*Lepisosteus oculatus* Prx1, XP_006634894.1), LoPrx2(*Lepisosteus oculatus* Prx2, XP_006631553.1), LoPrx3(*Lepisosteus oculatus* Prx3, XP_006630529.1), LoPrx4(*Lepisosteus oculatus* Prx4, XP_015196800.1), CmPrx1(Callorhinchus milii Prx1, NP_001279683.1), CmPrx2(Callorhinchus milii Prx2, NP_001279497.1), CmPrx3(Callorhinchus milii Prx3, XP_007903837.1), CmPrx4(Callorhinchus milii Prx4, NP_001279887.1), PmPrx2(*Petromyzon marinus* Prx2, ENSPMAG00000007606, Ensembl) and PmPrx3(*Petromyzon marinus* Prx3, ENSPMAG00000001457, Ensembl). Amino acid sequences of *Branchiostoma belcheri* were collected from its genome database(http://genome.bucm.edu.cn/lancelet/index.php) as follows: BbPrx1(Bb_078400F), BbPrx3(Bb_135520R) and BbPrx4(Bb_103880R).

Analysis of the EST sequences obtained from *Branchiostoma belcheri tsingtaunese* ovary cDNA library revealed one clone with a significant homology to the Family 1,2 Prx sequence. The full-length cDNA clone consisted of 1108 nucleotides and encoded a 198 amino acid peptide (Fig 1A). Translation started at the first ATG codon with a stop codon (TAA) at the -9 to -7 position.

The deduced amino acid sequence had predicted mass of 22,150 Da and pI of 7.30. No cleavable signal sequence was present at its N-terminus, and no glycosylation site was found. It was a cytoplasmic protein as predicted by PSORT II (http://www.cbs.dtu.dk/services/NetNGlyc).

A protein database search was performed using the BLASTX program of the National Center for Biotechnology Information (NCBI). The deduced protein sequence was 78% identical to that of *Homo sapiens* Prx 1, showing similarities (69–78% identity) to other Family 1,2 Prxs from different species, including human, mouse, rat, carp, newt, silkworm, mosquito, nematode and yeast (data not shown).

All proteins aligned with Bbt-TPx1 contained two highly conserved cysteines (Fig 1B), and are referred to as 2-cys peroxiredoxins. The two highly conserved cysteines were the peroxidatic cysteine (at position 52) and resolving cysteine (at position 173), and constituted redox-active sites. The presence of two conserved cysteines (at positions 52 and 173) suggested that this protein is a new member of the TPx family (in the peroxiredoxin superfamily) [14].

### Evolutionary analysis of Bbt-Tpx1

To further understand the relationships among Prx genes, more Prx amino acid sequences were used in phylogenetic analyses. A phylogenetic tree was constructed using the maximum likelihood method (Fig 1C). In this analysis, Bbt-Tpx1, Bb-Prx1 and a hypothetical Prx of *Branchiostoma floridae* (XP_002605995.1) were closely related to each other and together formed a separate branch in the subgroup of Prx1 and Prx2, whereas XP_002586269.1, another hypothetical Prx of *Branchiostoma floridae* was closely related to Bb-Prx4 and found to be group with other Prx4 of various species.

Taken together, it seemed that all Prx1 and Prx2 genes were derived from a common ancestor. Since lamprey Prx2 (PmPrx2) was found to group with previously reported Prx2 genes other than with Bbt-Tpx1, Bb-Prx1 or XP_002605995.1, the duplication of Prx1 and Prx2’s ancestor gene might happen after amphioxus emergence and before lampreys emergence. The lamprey Prx1 gene might be lost, whilst Prx1 of other species underwent a neofunctionalization. This finding corroborates the viewpoint that amphioxus is a transitional species between invertebrates and vertebrates.

### Expression profile by RT-PCR

**Fig 2:**
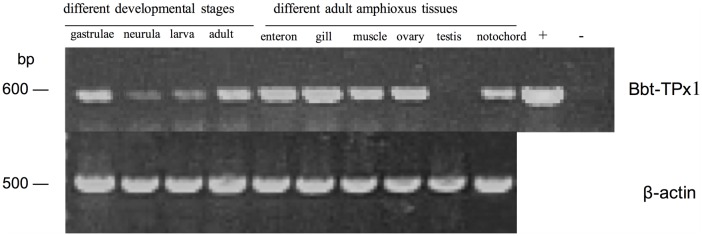
RT-PCR analysis of the Chinese amphioxus TPx1. Lanes 1–4: gastrulae, neurula, larva, adult, respectively (different developmental stages); lanes 5–10: enteron, gill, muscle, ovary, testis, notochord, respectively (different adult amphioxus tissues); lane 11: positive control (+) [Bbt-TPx1 amplified with pET32a(+)M-Bbt-TPx1]; lane 12: no template (-).

To assess the expression profile of Bbt-TPx1, RT-PCR was performed using various cDNA templates isolated from tissues of different developmental stages and adult amphioxus. As shown in Fig 2, Bbt-TPx1 was expressed in all developmental stages, with the highest level found in gastrulae. In addition, Bbt-TPx1 was expressed in all adult amphioxus tissues except testis, with the highest level found in the gill.

Because ovary and testis samples were obtained from mature adult female/male amphioxus, they were full of unfertilized eggs and sperms. High Bbt-TPx1 expression in the ovary was due to its high maternal levels in amphioxus, which increases gradually to peak in gastrulae before decreasing sharply in the subsequent developmental stages.

The gill is not the main respiratory organ in amphioxus, but the latter also breathes with the gill. TPx plays a role in protection against oxidative damage in living organisms. The highest expression level in the gill among adult amphioxus tissues supports the notion that Bbt-TPx1 might participate in innate immunity.

### Expression and purification of the recombinant Bbt-TPx1

**Fig 3:**
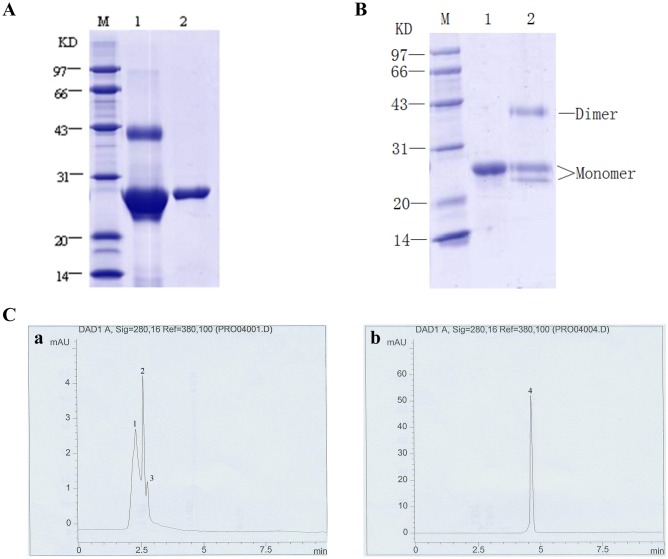
Properties of recombinant Bbt-TPx1. (A) SDS-PAGE (12% gel) analysis of rBbt-TPx1 in reducing conditions with Coomassie Brilliant Blue R-250 staining. Lane 1: rBbt-TPx1 purified by Ni^2+^ chelate column; lane 2: rBbt-TPx1 of lane 1 purified by SP-Sepharose column; lane M: molecular weight markers. (B) SDS-PAGE (12% gel) analysis of reduced and non-reduced rBbt-TPx1 samples. Lane 1: reduced rBbt-TPx1; lane 2: non-reduced rBbt-TPx1; lane M: molecular weight markers. (C) HPLC analysis of rBbt-TPx1 before (a) and after (b) treatment with 50 mM DTT for 12h.

The cDNA encoding Bbt-TPx1 was amplified using specific primers. The PCR product was cloned into the pET32a (+) M expression vector and expressed in *E*. *coli* as a histidine fusion protein. The rBbt-TPx1 protein was purified by Ni^2+^ chelate column, resulting in a >80% purity; the protein’s molecular mass was about 24 kDa according to SDS-PAGE (Fig 3A, Lane 1). The rBbt-TPx1 protein was further purified by SP-Sepharose column, and >99% purity was obtained (Fig 3A, Lane 2). The fusion protein showed a slightly larger size compared with the predicted molecular mass of 22.15 kDa, due to the N-terminal leader peptide of 2 kDa encoded by the expression vector.

### Properties of recombinant Bbt-TPx1 in reducing and non-reducing SDS-PAGE, and gel filtration chromatography

SDS-PAGE indicated that reduced rBbt-TPx1 had an apparent molecular mass of 24 kDa (Fig 3B, Lane 1). In non-reducing conditions, rBbt-TPx1 was visible as one broad band with a molecular mass of 42 kDa (Fig 3B, Lane 2), suggesting the presence of a dimer. The monomer appeared as the major band; a minor band was observed, probably representing molecules with different conformations [15]. Further analysis of rBbt-TPx1 was performed using HPLC gel filtration chromatography (Fig 3C). rBbt-TPx1 treated with DTT was eluted from an ODS-3 column as one discrete peak (4) (Fig 3C-b). However, rBbt-TPx1 without DTT treatment was eluted from the ODS-3 column as three discrete peaks (1)–(3) (Fig 3C-a). Peak (1) was most likely a dimer, while peaks (2) and (3) were monomers with different conformations.

Thus, both dimeric and monomeric forms were identified for the rBbt-TPx1 protein, and the dimer would be depolymerized into monomers with DTT. The rBbt-TPx1 protein had two highly conserved cysteines, Cys-52 and Cys-173. Cys-52 of one monomer attacked the Cys-173 of another monomer. This condensation reaction resulted in the formation of a stable inter-subunit disulfide bond, with rBbt-TPx1 subsequently forming a homodimer, which is reduced by one of several thiol disulfide reducers, to complete the catalytic cycle [16].

### Removal of H_2_O_2_ by recombinant Bbt-TPx1

**Fig 4:**
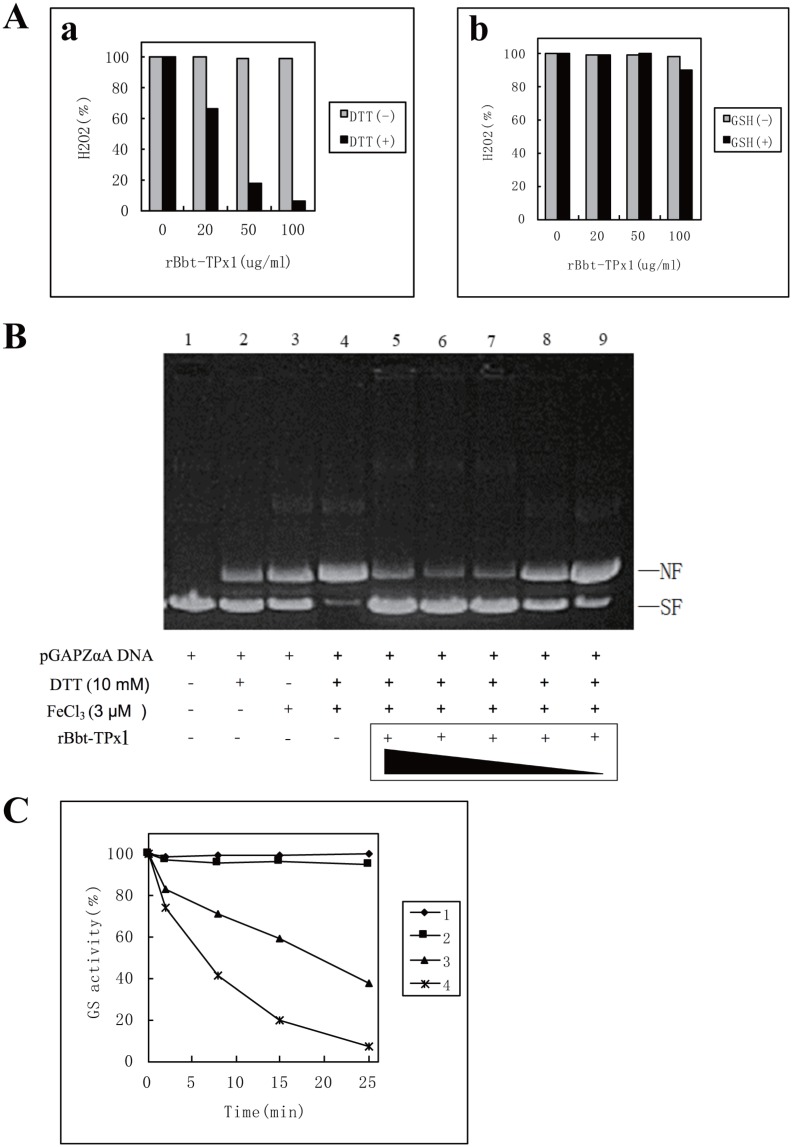
Antioxidant activity of rBbt-TPx1. (A) Removal of H_2_O_2_ by rBbt-TPx1 with DTT (a) or GSH (b). (B) rBbt-TPx1-dependent inactivation of DNA cleavage in the metal-catalyzed oxidation (MCO) system. Lane 1, pGAPZαA DNA; lane 2, pGAPZαA DNA and 10 mM DTT; lane 3, pGAPZαA DNA and 3 μM FeCl_3_; lane 4, pGAPZαA DNA, 10 mM DTT and 3 μM FeCl_3_; lanes 5–9: pGAPZαA DNA, 10 mM DTT, 3 μM FeCl_3_ with 500, 200, 100, 50, and 25 μg/ml rBbt-TPx1, respectively. NF: nicked form of the pGAPZαA plasmid DNA; SF: supercoiled form of the pGAPZαA plasmid DNA. (C) GS protection by rBbt-TPx1 against the DTT/Fe^3+^/O_2_ system. 1 mM EDTA; rBbt-TPx1 at 100 μg/ml, 20 μg/ml, or 0 μg/ml.

The antioxidant activity of rBbt-TPx1 in removing H_2_O_2_ was evaluated using the ferrithiocyanate system. The rBbt-TPx1 protein had this property in the presence of DTT (Fig 4A-a). Within 10 min the 100 μg/ml solution of rBbt-TPx1 destroyed about 98.5% of 200 μM H_2_O_2_. H_2_O_2_ removal was rBbt-TPx1 protein concentration dependent. However, rBbt-TPx1 lost this capability in the absence of DTT (Fig 4A-b). When DTT was replaced with GSH, rBbt-TPx1 failed to remove H_2_O_2_, suggesting that DTT is capable of reducing the oxidized form of rBbt-TPx1, regenerating its enzymatic activity, but not GSH. Combined with the above findings in non-reducing SDS-PAGE, it could be deduced that rBbt-TPx1 generally exists as an inactive dimer. The dimer has two inter-molecular disulfide bonds, and requires two thiol donor reducers, such as DTT, to yield the active monomer. However, one thiol donor reducers, such as GSH, activity is not obtained.

The rBbt-TPx1 protein might have three forms: oligomer (data not shown), dimer and monomer. Oligomerization has been reported for a number of peroxiredoxins, in particular yeast TPx [3], the Escherichia coli AhpC C-terminus [17] and pea mitochondrial PsPrxII F [18]. Inactivity and activity of the rBbt-TPx1 protein might change with its form. The protein formed a redox cycle by DTT and H_2_O_2_ *in vitro*. However, the link between the oligomeric state and function of TPx remains unclear.

### Metal-catalyzed oxidation

To further evaluate the antioxidant activity of rBbt-TPx1, its protective effects on DNA and protein were assessed.

The hydroxyl radicals produced in the MCO system can cause nicks in supercoiled plasmids, and the DNA can be protected by TPx [4]. The extent of DNA damage was evaluated by assessing the shift in gel mobility of pGAPZαA as it was converted from the supercoiled to the nicked form. In the absence of the rBbt-TPx1 protein, the ROS produced in the MCO system caused nicking of the supercoiled pGAPZαA DNA (Fig 4B, Lane 4). In the same reaction mixture without the MCO system, pGAPZαA remained supercoiled under the same condition (Fig 4B, Lane 1). This indicated that pGAPZαA DNA conversion from the supercoiled to the nicked form is absolutely dependent on MCO system-induced oxidative stress. Addition of purified rBbt-TPx1 at concentrations between 25 and 500 μg/ml to the MCO system prevented nicking of the supercoiled DNA by the ROS generated in the assay (Fig 4B, Lane 5–9), in a dose dependent manner. This finding indicated that the rBbt-TPx1 protein protects DNA from damage by the MCO system: the higher the rBbt-TPx1 concentration, the greater the protection, corroborating previous data. MCO system produced ROS could also destroy the activity of thiol proteins such as glutamine synthetase (GS) [3], which could be protected in the presence of rBbt-TPx1. Under the assay conditions described in Materials and Methods, about 93% glutamine synthetase activity was lost after 25 min in the absence of a protector (Fig 4C). Addition of 1 mM EDTA completely prevented GS inactivation by the DTT/Fe^3+^/O_2_ system (Fig 4C). Fig 4C shows the time-dependent inactivation of GS by the DTT/Fe^3+^/O_2_ system at different rBbt-TPx1 protein concentrations. Incubation for 25 min caused 5% and 63% GS inactivation in the presence of 100 and 20 μg/ml rBbt-TPx1, respectively (Fig 4C).

Our results indicated that rBbt-TPx1 protected DNA and various enzymes from damage caused by the DTT/Fe^3+^/O_2_ system *in vitro*, but it requires further study to show if this protection relies on the enzymatic activity of rBbt-TPx1. However, the *E*. *coli* catalase destroying H_2_O_2_ faster than rBbt-TPx1 (data not shown) did not show the ability to protect DNA and various enzymes from damage caused by the DTT/Fe^3+^/O_2_ system *in vitro*. Our findings suggest that Bbt-TPx1 plays an antioxidant role *in vivo* and helps cells survive the oxidative stress.

## Abbreviations

DTT: dithiothreitol
GS: glutamine synthetase
GSH: glutathione
H_2_O_2_: non-radical hydrogen peroxide
HO: hydroxyl radicals
LB: Luria broth
MCO: metal-catalyzed oxidation
NCBI: National Center for Biotechnology Information
O2 −: superoxide anions
Prxs: Peroxiredoxins
ROS: Reactive oxygen species
RT-PCR: Reverse transcription polymerase chain reaction
TPx: thioredoxin peroxidase
TR: thioredoxin reductase
